# Placenta Extending From the Anterior to the Posterior Uterine Wall: A Rare Case of Pregnancy With a Partial Septate Uterus

**DOI:** 10.7759/cureus.88124

**Published:** 2025-07-16

**Authors:** Naomi Ohta, Keita Hasegawa, Kaori Suenaga, Yoshiyuki Mochimaru, Toru Arase

**Affiliations:** 1 Department of Obstetrics and Gynecology, Keiyu Hospital, Yokohama, JPN

**Keywords:** differential diagnosis, placenta, septate uterus, uterine anomaly, uterine cavity

## Abstract

Placental attachment spanning from the anterior to the posterior uterine wall, resulting in the formation of two uterine cavities, presents a diagnostic challenge in obstetrics. Differential diagnoses include placental abruption, multiple gestations, placental variations, and uterine anomalies. Accurate prenatal identification is often difficult, especially when anomalies are not detected during early pregnancy. We report a rare case of a 27-year-old primigravid Japanese woman with a placenta spanning from the anterior to the posterior uterine wall, creating two cavities. At 35 weeks of gestation, ultrasound suggested an unusual placental position, with fetal parts located in separate cavities. At 37 weeks and three days, a cesarean section was performed for breech presentation. Intraoperatively, a partial uterine septum was palpated postplacental removal, with a planar fundus and no retroplacental hematoma. Postoperative ultrasound confirmed the diagnosis of a partial septate uterus. Although septate uterus is the most common uterine anomaly, it often remains undiagnosed in women without infertility. This case highlights the importance of considering uterine anomalies when placental morphology is atypical, especially in the absence of a prior diagnosis, as early recognition is crucial for appropriate perinatal management.

## Introduction

During pregnancy, it is important to assess the placental position and morphology, as these factors influence the mode of delivery and are associated with various complications, such as abnormal bleeding and fetal growth restriction [1]. When an obstetrician encounters a placenta that extends from the anterior to the posterior uterine wall and creates two cavities, the differential diagnoses may include placental abruption, multiple pregnancies, placental variations, or uterine anomalies. One type of placental variation is the bilobate placenta, in which two placental lobes are present, with an incidence of up to 4% [2]. Previously, H-shaped bilobate placental partitions have been reported, and this morphological anomaly has been suggested as a possible factor associated with fetal growth restriction [3].

Among congenital uterine anomalies, a septate uterus, a condition in which a septum divides the uterus into two cavities, is the most common and is associated with adverse obstetric outcomes, including miscarriage, preterm birth, malpresentation, fetal growth restriction, and placental abruption [4-6]. However, unless it has been previously diagnosed, typically in the context of infertility or recurrent pregnancy loss, a septate uterus may go undetected until delivery or cesarean section. This is particularly true in cases where the patient has conceived spontaneously, and no abnormalities are noted in routine prenatal care. In such cases, unusual placental morphology may raise concerns about fetal well-being and delivery planning, especially when imaging does not provide a definitive diagnosis.

Here, we report a rare case of a placenta spanning the uterine walls, creating two cavities, which was diagnosed as a partial septate uterus during a cesarean section. This case highlights the importance of considering uterine anomalies in the differential diagnosis of unusual placental presentations and underscores the need for careful prenatal morphological assessment, even in patients without known risk factors.

## Case presentation

**Figure 1:**
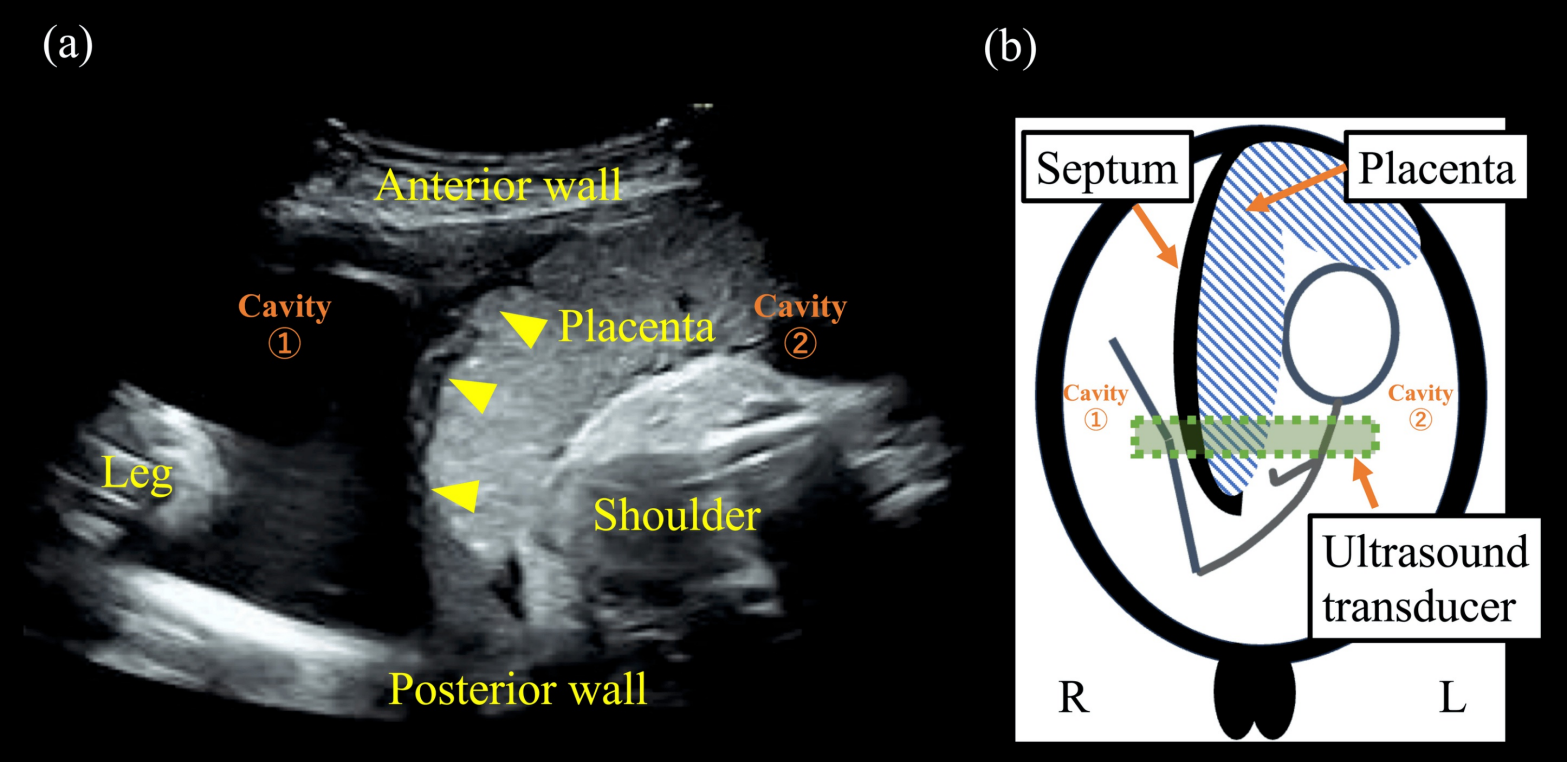
Transabdominal ultrasonography image at 35 weeks of gestation (a) Ultrasonographic findings at 35 weeks’ gestation show the placenta attached from the anterior to the posterior wall of the uterus, dividing it into two cavities. A fetal leg and shoulder are observed in the cavities. One side of the placenta appears gently elevated, whereas the other side, attached to the septum, is compressed (arrowhead). (b) The positions of the fetus, septum, and placenta are indicated in the schematic. This figure was created by the authors

The patient was a 27-year-old primigravid Japanese woman (gravida 1, para 0) who conceived naturally and was referred for a singleton delivery. She underwent regular antenatal examinations, during which no abnormalities were identified. At 35 weeks of gestation, ultrasonography showed the placenta extending from the anterior to the posterior wall, with fetal parts in the cavities (Figure 1).

An internal examination confirmed the presence of a single cervix and vagina. The nonstress test (NST) showed a baseline fetal heart rate of approximately 140 bpm with moderate variability and accelerations, without decelerations, indicating a reactive pattern. As fetal well-being and growth were confirmed and no abnormalities, such as retroplacental hematoma, were found, outpatient monitoring was continued. The estimated fetal weight remained within the normal range throughout the pregnancy. The patient had an uneventful pregnancy without any notable symptoms, and a cesarean section was performed at 37 weeks and three days of gestation due to breech presentation. Intraoperatively, after placental removal, the uterine septum was palpated, and the fundus was found to be planar. No retroplacental hematomas were observed. The newborn weighed 2,300 g, with Apgar scores of 9 and 10 at one and five minutes, respectively, and an umbilical artery pH of 7.28. 

**Figure 2:**
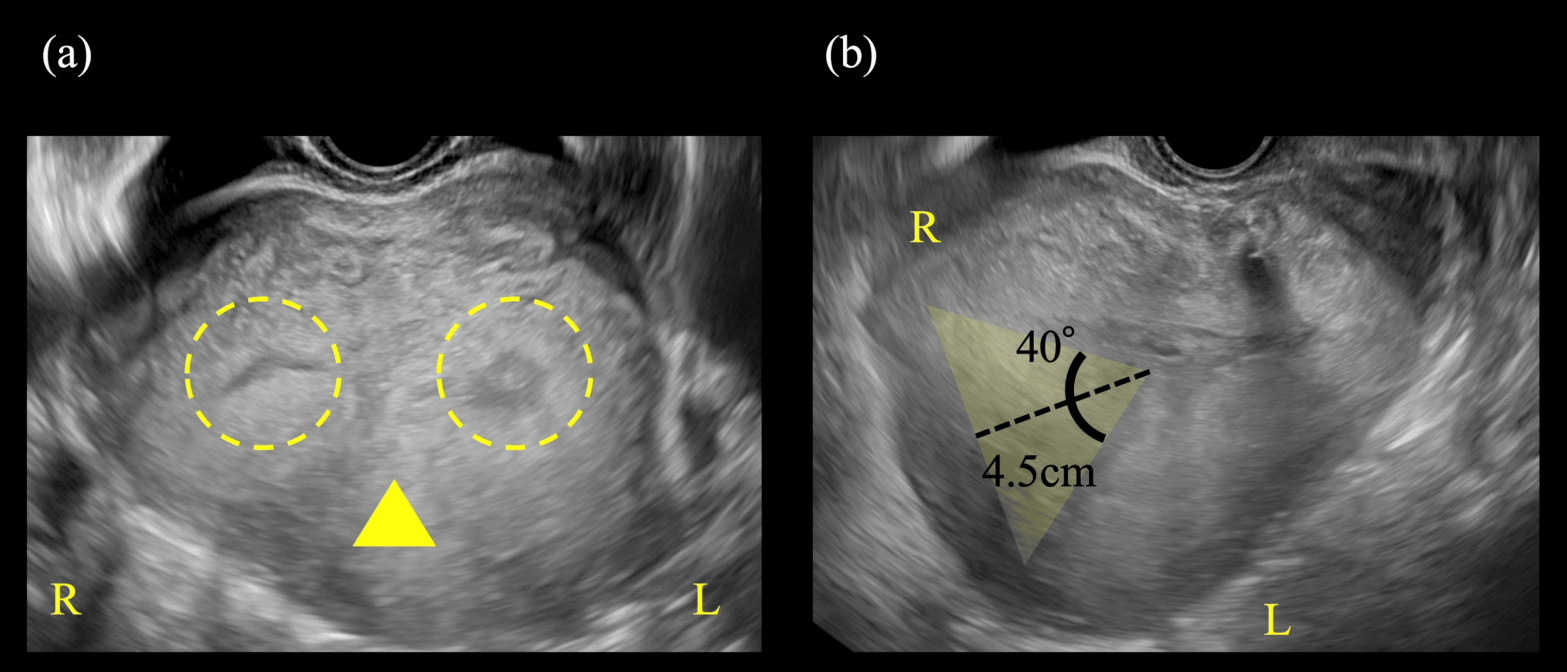
Predischarge transvaginal ultrasonography image of the uterus (a) Predischarge ultrasonography image of the uterus shows a septum (arrowhead) dividing it into two cavities (dotted circle). (b) The uterine fundus has a planar shape. The septum measured 4 cm in length with a septal angle of 40°, and no serosal indentation was observed

After surgery, she had an uncomplicated course, and the newborn required no specific interventions. One week postoperatively, ultrasonography confirmed an incomplete septum with a planar fundus. Therefore, a partial septate uterus was diagnosed (Figure 2).

## Discussion

To the best of our knowledge, this is among the first reports to present an image showing placental attachment to the uterine septum during pregnancy. In addition, due to the rarity of this condition, it is important to consider a differential diagnosis, including potentially life-threatening conditions (Table 1).

**Table 1:** Differential diagnoses for a placenta spanning the uterine cavity

Category of origin	Differential diagnoses
Placental problem	Placental abruption, placental variation (e.g., bilobed placenta)
Fetal problem	Multiple pregnancy
Uterine problem	Uterine anomaly (e.g., septate uterus)

When an obstetrician encounters a placenta that extends from the anterior to the posterior uterine wall and creates two cavities, placental abruption should first be excluded. Multiple pregnancies should be considered if multiple fetuses are present. Uterine anomalies, such as a septate or bicornuate uterus, should be suspected. If both sides of the placenta are gently elevated, a bilobate placenta is possible. A uterine anomaly should be suspected if one side is compressed (Figure 1).

Although the septate uterus is the most common uterine anomaly, the prevalence is estimated to be only one to two per 1,000 women and as high as 15 per 1,000 women [7]. It is associated with complications, such as spontaneous abortion, preterm birth, placental abruption, and malpresentation [4,5]. These complications may result from narrowing of the uterine cavity and associated vascular insufficiency [8,9]. In the present case, the infant was classified as having low birth weight. The most common diagnostic method is magnetic resonance imaging (MRI). Distinguishing between a septate uterus and a bicornuate uterus is important for selecting surgical methods; however, MRI is costly and may pose a significant burden to patients. Several studies have reported treatment outcomes for a septate uterus, with a live birth rate of 81.3% in the surgical treatment group and 61.5% in the nonsurgical treatment group [10]. Therefore, if this condition is not diagnosed before pregnancy, especially in patients without infertility, it may be difficult to identify during pregnancy. Moreover, it is important to assess not only intrauterine contents such as the gestational sac and fetus, but also the overall uterine morphology during the first trimester.

## Conclusions

We report a rare case of a placenta dividing the uterine cavity, which was ultimately diagnosed as a partial septate uterus. When an obstetrician encounters a placenta that extends from the anterior to the posterior uterine wall and creates two cavities, differential diagnoses should include emergent conditions, such as placental abruption and uterine anomalies. Since this condition may be associated with malpresentation or small-for-gestational-age fetuses and requires careful management, early identification of uterine anomalies is crucial. At the first outpatient visit, assessment of both uterine morphology and gestational tissues is important, especially in patients without known infertility. Increased awareness of such atypical placental morphology may lead to earlier diagnosis and improved prenatal care. In addition, identifying such anomalies during pregnancy may assist in surgical planning and inform counseling regarding future pregnancies, particularly in terms of delivery mode and risk management. Further studies are needed to better understand the clinical implications and optimal management strategies for such rare uterine anomalies.
